# Efficacy and safety of ivermectin and albendazole co-administration in school-aged children and adults infected with *Trichuris trichiura*: study protocol for a multi-country randomized controlled double-blind trial

**DOI:** 10.1186/s12879-019-3882-x

**Published:** 2019-03-18

**Authors:** Chandni Patel, Eveline Hürlimann, Ladina Keller, Jan Hattendorf, Somphou Sayasone, Said M Ali, Shaali M Ame, Jean T Coulibaly, Jennifer Keiser

**Affiliations:** 1 Swiss Tropical and Public Health Institute, Basel, Switzerland; 2 University of Basel, Basel, Switzerland; 3 Lao Tropical and Public Health Institute, Vientiane, Lao People’s Democratic Republic; 4 Public Health Laboratory Ivo de Carneri, Chake Chake, Zanzibar, Pemba Tanzania; 5 Unité de Formation et de Recherche Biosciences, Université Félix Houphouët-Boigny, Abidjan, Côte d’Ivoire; 6 Centre Suisse de Recherches Scientifiques en Côte d’Ivoire, Abidjan, Côte d’Ivoire

**Keywords:** *Trichuris trichiura*, Côte d’Ivoire, Lao PDR, Tanzania, Drug efficacy, Ivermectin, Albendazole, Soil-transmitted helminthiasis, Drug safety

## Abstract

**Background:**

Soil-transmitted helminthiasis affects almost 2 billion people worldwide in tropical climates. Preventive chemotherapy, using the benzimidazoles (albendazole and mebendazole) is the current main recommended control strategy. Nevertheless, there is limited efficacy of these drugs against hookworm infection and, to a greater extent, against trichuriasis. We describe a protocol for a trial investigating the efficacy and safety of the co-administration of ivermectin and albendazole against trichuriasis.

**Methods:**

A double-blind, placebo-controlled randomized controlled trial will be conducted in three countries (Côte d’Ivoire, Tanzania and Lao PDR) with the aim to determine the efficacy, safety and extended effects of co-administered ivermectin and albendazole compared to standard albendazole monotherapy. We will enroll 600 participants aged 6–60 years in each setting. The primary outcome is cure rate (CR) against *Trichuris trichiura* infection as assessed by Kato-Katz 14–21 days after treatment. Secondary outcomes include CRs against concomitant soil-transmitted helminth (STH) infections (*Ascaris lumbricoides*, hookworm and *Strongyloides stercoralis*) and egg reduction rates (ERRs) against STH at 14–21 days, 180 days and 360 days. Tolerability of treatment, infection status assessed by polymerase chain reaction (PCR), and potential benefits of deworming on nutritional and morbidity indicators will be assessed. The primary analysis will include an available-case set and use logistic regression models adjusted for age, sex and weight.

**Discussion:**

This trial will provide robust results on the efficacy and safety of co-administration of ivermectin and albendazole with the aim to better inform WHO recommendations on control of STHs. Furthermore, secondary and explanatory outcomes will provide direct evidence on the extended effects of combination therapy and insight on the relationship between nutrition and morbidity parameters and infection status and intensity.

**Trial registration:**

NCT03527732 (date assigned: 17 May 2018).

**Electronic supplementary material:**

The online version of this article (10.1186/s12879-019-3882-x) contains supplementary material, which is available to authorized users.

## Background

Almost 2 billion people are infected with soil-transmitted helminths (STHs), the majority being preschool and school-aged children living in Asia and Africa [1, 2]. *Ascaris lumbricoides* (roundworm), *Trichuris trichiura* (whipworm) and *Necator americanus* or *Ancylostoma duodenale* (hookworms), the most common STHs, account for an annual burden of 1.9 million disability-adjusted life-years (DALYs) related to infection [1, 3]. Infection can be the result of the ingestion of *T. trichiura* or *A. lumbricoides* eggs or the penetration of skin by hookworm larvae [4]. Inadequate access to clean water, poor hygiene and unimproved sanitation lead to an increase in risk of STH infection, thus particularly affecting populations in low- and middle-income countries [4]. Morbidity due to STHs are related mostly to high intensity infections and may include acute symptoms such as diarrhea, dysentery, abdominal pain or obstruction; if left untreated, STH infections can lead to inflammation, nutrition and immune system impairment and, finally, can cause physical and mental development retardation in children and limited working capacity in adults [1, 4].

To date, preventive chemotherapy (PC) that is the periodic administration of anthelminthic drugs to at-risk populations without prior diagnosis, is the cornerstone of helminth control put forth by the World Health Organization (WHO) to reduce the burden of STHs. PC is implemented in the form of annual mass drug administration (MDA) campaigns and the recommended target populations have been expanded from only school-aged children to include younger children (1–5 years of age), adolescent (10–19 years) girls, women of reproductive age (15–49 years) and pregnant women after the first trimester in areas with an STH prevalence of ≥20% [5]. A biannual frequency of MDA is recommended in case of high prevalence (> 50%). Albendazole, one of the two main drugs used for MDA, is considered safe and well tolerated; however, it is not efficient in clearing infection and reducing worm loads in all three types of STHs [2, 6]. While it shows satisfyingly high cure rates (CRs) (96%) and egg reduction rates (ERRs) (> 98%) against *A. lumbricoides*, efficacy against hookworm is lower (CR = 80% and ERR = 90%) and against *Trichuris trichiura* is disturbingly low with CRs of 31% and ERRs of 50% [6]. Furthermore, this drug has been used for more than three decades and comparison of efficacy measures over time indicates a decreasing trend over time; although resistance has so far not been documented in its use in humans [5]. In view of insufficient efficacy, especially against *T. trichiura*, coupled with the potential for resistance emergence from long-term use, there is not only a pressing need for the development of new treatments against STH infections, but also a need to optimize current treatment schemes [7].

The co-administration of standard drugs together with other anthelminthics, such as ivermectin, could be a way to achieve universal impact on all STH species [8]. Moreover, the combined use of ivermectin and albendazole against STH infection has been added recently to the WHO Model Lists of Essential Medicines paving the way for application in control programs [9]. A recent meta-analysis indeed shows a lower risk (risk ratio (RR) =0.44) of still being positive for *T. trichiura* post-treatment for co-administered ivermectin and albendazole when compared to albendazole alone; however, these findings are based on a very limited amount of qualifying studies (*n* = 3) conducted in various settings, limited to school-aged children and/or adolescents with considerable variation among the reported efficacy measures [10]. Interestingly, two studies also highlight potential extended effects on follow-up infection status or intensity (18 weeks and 1 year) compared to single or other combined drug regimens [11, 12]. To better inform and guide ongoing helminth control programs on optimization and implementation of ivermectin-albendazole integrated treatment schemes, a deeper understanding of its impact across a range of different transmission settings, including a broader age range and a prolonged monitoring period, is needed.

To the best of our knowledge, this is the first large, multi-country trial assessing the safety and efficacy of the combination of ivermectin and albendazole and the extended effects of treatment against *T. trichiura*.

## Methods/design

### Trial design

**Fig. 1:**
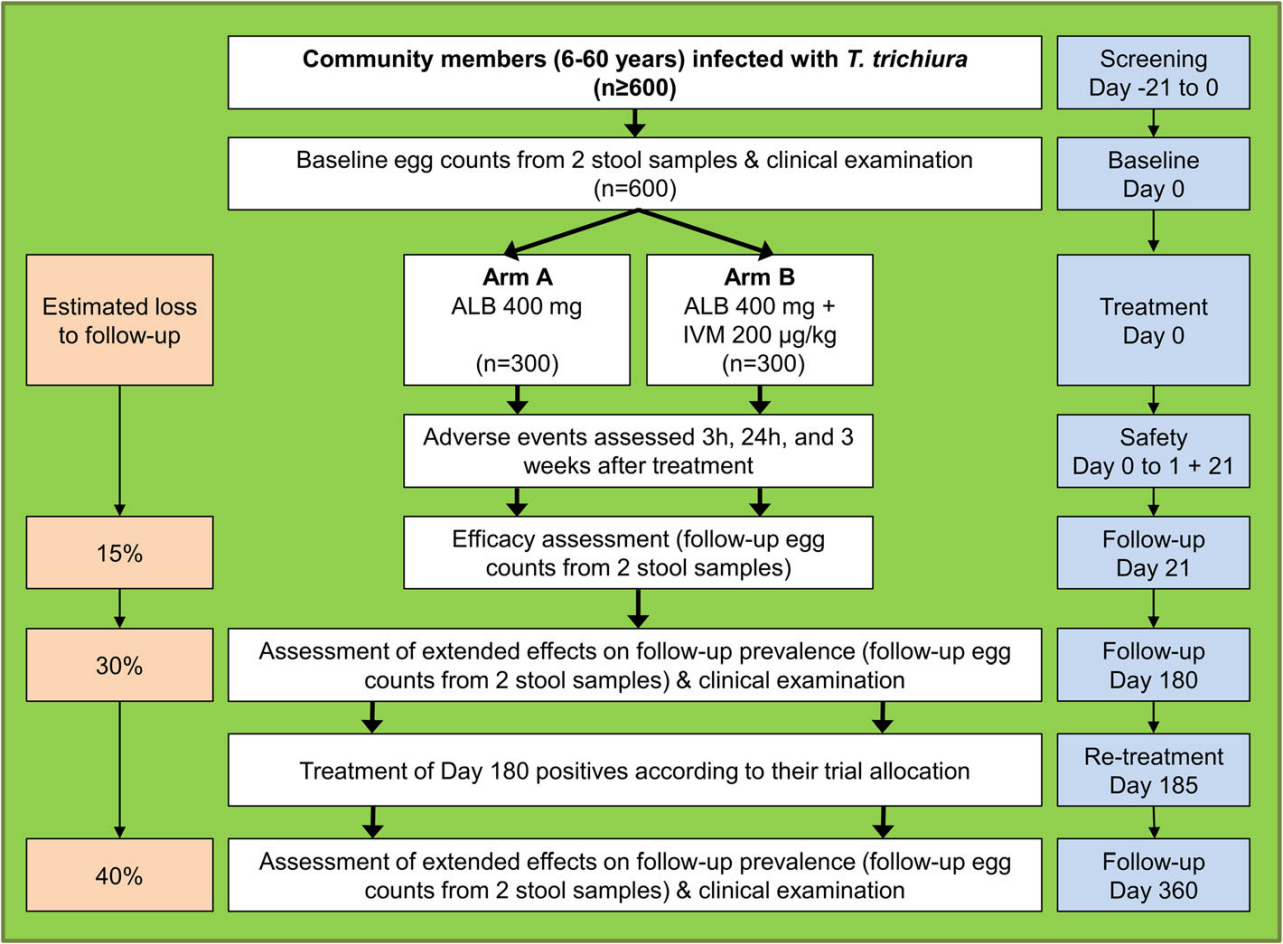
Design and timeline of the randomized controlled trial to be implemented in each of three settings. The study is designed as a two-armed trial including one arm with a single drug administration (arm A; albendazole) and one arm with combined treatment through co-administration of separate tablets (arm B; ivermectin and albendazole). The trial will be conducted as a multi-country study with two settings in Africa and one in Asia, namely Côte d’Ivoire, Pemba (Zanzibar, Tanzania) and Lao PDR

This is a multi-country, parallel group, double-blind, placebo-controlled, randomized controlled trial (RCT). It will be implemented as a community-based study targeting children, adolescents and adults aged 6 to 60 years. In each of the three countries, 600 *T. trichiura*-positive community members will be randomly assigned to either receive the current standard treatment (albendazole/placebo) or the combination therapy (ivermectin/albendazole). All participating communities will be followed-up over a period of one year including four assessment time points at baseline, 3 weeks, 6 months and 12 months after treatment (see Fig. 1). The WHO Trial Registration Data Set summarizes the most important trial information and is given in Additional file 1. The study adheres to the Standard Protocol Items: Recommendations for Interventional Trials (SPIRIT) statement [13] and a checklist is provided as supplementary file (see Additional file 2).

### Study area and participants

The trial will be conducted in two African settings, namely Côte d’Ivoire and Pemba Island, Tanzania, and one Asian setting, Lao People’s Democratic Republic (PDR). Potential study areas will be selected based on earlier findings and insights from local collaborators on *T. trichiura* endemicity. In Côte d’Ivoire, the Agnéby-Tiassa region in the southeast of the country has been identified with high STH infection prevalence (particularly for *T. trichiura*) in previous community-based studies or RCTs [14, 15]. Pemba Island is still highly endemic to STH infections [16]. Potential study communities on Pemba will be selected based on accessibility besides *T. trichiura* endemicity. An area fulfilling these criteria is considered the Shehia of Pujini, 9 km south-east of Chake Chake, Pemba. In Lao PDR recent data suggests highest infection rates for *T. trichiura* to be found in the northern zone of the country including the province of Luang Prabang [17]. In each study location community members from 6 to 60 years of age will be invited for participation.

### Recruitment

In order to recruit participants from a broad age range (6–60 years), entire communities with less than 1000 inhabitants (smaller communities are easier to be mobilized and monitored) will be pre-screened and a census conducted to identify *T. trichiura* cases and eligible individuals in each household, respectively.

All adult community members will be invited to participate in an informational meeting explaining the purposes and procedures of the study, including potential benefits and risks. In this open discussion forum, parents/caregivers/potential participants will be encouraged to ask questions and be informed of actions to prevent acquiring STH infections in the future.

Individuals (including parents/caregivers of children) interested in participating in the trial will be invited to complete the process of informed consent; thereafter, individuals will be assessed for study eligibility during screening procedures.

### Eligibility criteria

Participants will be eligible to be included in the trial if they fulfill all of the following criteria:

1. Provide written informed consent signed by either the participant him/herself (≥18 years of age in Lao PDR and Pemba, Tanzania or ≥ 21 years of age in Côte d‘Ivoire) or by parents and/or caregivers for children/adolescents; and oral assent by child/adolescent (aged 6–17 years Lao PDR and Pemba, Tanzania or aged 6–20 years in Côte d‘Ivoire).
2. Agree to comply with study procedures, including provision of two stool samples at the beginning (baseline) and on three follow-up assessments (approximately 3 weeks, 6 months, and 12 months later).
3. Aged ≥6 to ≤60 years and weighing at least 15 kg.
4. Positive for *T. trichiura* infection in at least two slides of the quadruple Kato-Katz thick smears and infection intensity of at least 100 eggs per gram (EPG) of stool.

Participants will be ineligible to be included in the trial if they fulfill any of the following criteria:

1. Presence of major systemic illnesses, e.g. severe anemia (below 80 g/l Hemoglobin (Hb) according to WHO [18]), clinical malaria as assessed by a medical doctor (positive *Plasmodium* rapid diagnostic test (RDT) and ≥ 38 °C ear temperature), upon initial clinical assessment.
2. History of acute or severe chronic disease (e.g. cancer, diabetes, chronic heart, liver or renal disease).
3. Recent use of anthelmintic drug (within past 4 weeks).
4. Attendance in other clinical trials.
5. Known allergy to study medications (i.e. ivermectin and albendazole).
6. Pregnancy or lactating in the 1st week after birth (according to WHO guidelines within lymphatic filariasis control programs [19]).
7. Current use of medication with known interaction (e.g. for ivermectin: warfarin; for albendazole: cimetidine, praziquantel and dexamethasone).

### Intervention

All *T. trichiura*-infected, consenting, and participating community members will be treated with the respective single or combination treatment regimen at day 0. Re-treatment with the intervention assigned at randomization will occur at 6 months in all participants found to be positive for any STH*.* 400 mg albendazole tablets will be the product of Glaxo Smith Kline, UK (Zentel®) and a single tablet will be administered. 3 mg tablets of ivermectin (Stromectol®) will be obtained from Merck, France and administered at a dose of 200 μg/kg body weight recorded for each participant. Matching ivermectin placebo tablets (in terms of appearance) will be produced and a certificate of manufacture and analysis be provided by the University of Basel. Since ivermectin and albendazole are known to be better absorbed in humans after a high-fat meal is consumed, participants will receive a local high-fat breakfast prior to treatment [20, 21]. After ingestion of the medication, the subjects will be observed for 3 h to ensure retention of the drug. Vomiting within 1-h post-dosing will require re-dosing. The subjects will not be allowed more than one repeated dose. No re-administration will be needed for subjects vomiting after one hour.

### Outcomes

#### Primary outcome

The primary outcome is *T. trichiura* infection status as assessed by Kato-Katz 14–21 days after treatment measured as CR, calculated as the percent of infected individuals at baseline free from infection after treatment.

#### Secondary outcomes

Secondary outcomes include the ERR against *T. trichiura*, CRs and ERRs against other concomitant STH infections (*A. lumbricoides*, hookworm and *Strongyloides stercoralis*), reinfection rates, tolerability of treatment and infection status assessed by polymerase chain reaction (PCR). Outcomes will be assessed at 14–21 days, 180 days and 360 days post-treatment.

#### Exploratory outcomes

Exploratory outcomes include the molecular characterization of *T. trichiura* strains from different settings and investigation of potential resistance markers through deep sequencing as well as the evaluation of the potential benefits of deworming on nutrition status and morbidity indicators.

#### Sample size calculation

Based on a recent systematic review and the published literature, we assume that the CR of albendazole against *T. trichiura* is 30% compared to 50% in the ivermectin-albendazole treatment regimen [6]. To achieve a power of 90% at a significance level of 5%, 121 participants per study arm are needed to detect a statistically significant difference. With an estimated loss to follow-up of 15%, 143 participants will be required in each study arm. Furthermore, we assume the same treatment efficacy and a reinfection risk of 10% at 6 months. Consequently we expect a proportion of negative patients after 12 months of 44% in the albendazole arm and of 65% in the ivermectin-albendazole arm resulting in a required sample size of 111 participants per arm. To account for a loss to follow-up of 30% after 6 months and 40% at final assessment (12 months), we aim to recruit 300 participants in each treatment group (600 in total) in each country for a total of 1800 participants.

#### Randomisation

Study participants eligible for treatment will be randomly assigned in a 1:1 allocation to one of the treatment arms using sealed, opaque sequentially-numbered envelopes prepared by persons independent of the trial. Since treatment success is influenced by infection intensity, stratified block randomization will be used (baseline infection intensity: light infections and moderate/heavy infections) to ensure balanced treatment groups in terms of infection intensity. The computer-generated stratified randomization sequence, provided by a statistician, will vary randomly in blocks of four, six and eight and will be stratified by 2 levels of baseline infection intensity (light: < 1000 EPG, and moderate and heavy: ≥1000 EPG *T. trichiura* infections).

#### Blinding

The trial will be double-blinded (i.e. study participants and the trial team/researchers conducting the treatment and assessing the outcomes will be blinded). One 400 mg albendazole tablet will be given to each participant. The ivermectin (or corresponding placebo) tablets used for each treatment arm will be repacked into neutral separate plastic bags each containing the maximum number of ivermectin tablets with regard to weight and dose or the corresponding number of appearance-matched placebo tablets produced by the University of Basel. If at any point during the trial an unanticipated need to unblind a participant’s treatment allocation arises for reasons of safety, the principal investigator, site investigators, and ethics committee will be notified and the instance will be documented.

#### Trial timeline

The trial will last for fourteen months. The screening for the baseline will start three weeks prior to the treatment. Follow-up screening will take place 14–21 days, 180 days and 360 days post-treatment and each will last for about three weeks. Schedules of visits are summarized in Table 1.

**Table 1:** Schedule of visits in trial

	Screening	Baseline/Treatment/Safety				Follow-up			
	-21 to -1 days	Hours				Days			
		0		3	24	21	180	185	360
Diagnosis (stool and urine examination)	X					X	X		X
Gut morbidity (stool RDTs)	X					X	X		X
Informed consent	X								
Demographics	X								
Medical history		X							
Clinical examination		X					X		X
Pregnancy testing		X					X		X
Hemoglobin measurement		X					X		X
*Plasmodium* co-infection (in Côte d’Ivoire/ Lao PDR only)		X					X		X
*W. bancrofti* co-infection (in Africa only)		X					X		X
Venous blood examination (in Côte d’Ivoire/ Lao PDR only)		X				X	X		X
Physical functioning		X					X		X
Randomization and treatment			X						
Selective re-treatment								X	
Capturing AEs				X	X	X			
Capturing SAEs				X	X	X			
Treatment satisfaction				X		X	X		

### Data collection

All data besides a household questionnaire will be collected during scheduled visits and recorded on paper case report forms (CRFs), laboratory reporting forms or logs. Subsequently, data will be double-entered into a database using EpiInfo (v3.5.4). Access will be limited to study investigators and study personnel entering data; both working independently from the project funder. Data reported in the household questionnaire will be collected using paper forms in Pemba Island and using Open Data Kit (ODK) on mobile tablet computer devices in Côte d’Ivoire and Lao PDR where electronic data collection has already been applied in earlier studies. Data entered via ODK collect will be uploaded to a server hosted by the Swiss Tropical and Public Health Institute (Swiss TPH; Basel, Switzerland). For quality assurance in-built error, range and consistency checks will be programmed for the data entry masks (i.e., in EpiInfo and ODK). The obtained data will be handled strictly confidentially. Personal data will be coded for data analysis. No names will be published at any time, and published reports will not allow for identification of single subjects.

### Clinical assessment

A clinical examination of the study participants assessing general health (blood pressure, pulse rate, symptoms, medical history, etc.), anthropometric parameters including height, weight, mid-upper arm circumference (MUAC) and skinfold thickness (i.e. tricep and subscapular skinfolds) as well as tympanic temperature using an ear thermometer (Braun Thermoscan 5, Braun GmbH, Kronberg, Germany) will precede the treatment and will be repeated on two follow-up assessments (days 180 and 360) to evaluate potential benefits from deworming. Blood pressure will be measured using a sphygmomanometer (OMRON M6, Omron Healthcare CO., LTD, Kyoto, Japan). Body weight will be measured using a mobile, digital scale (SECA Model 803, Seca Gmbh Co, Hamburg, Germany) with a precision to the nearest 0.1 kg, while height will be measured using a measuring stick in centimetres. Mid-upper arm circumference will be measured to the nearest 0.1 cm using a MUAC tape; and a caliper (Harpenden Skinfold Caliper, HaB International Ltd., Warwickshire, England) will be used to measure tricep and subscapular skinfold thicknesses to the nearest 0.2 mm. All anthropometric measurements will be taken twice, recorded and the average value then used. A licensed physician will conduct a physical examination on each participant before treatment at baseline, 6 months and 12 months.

### Biospecimen collection and testing

#### Stool samples

Community members providing informed consent will be asked to provide two stool samples of at least 15 g each within a maximum of 5 days at baseline. From every stool specimen, duplicate Kato-Katz thick smears (41.7 mg each) will be prepared and read under a microscope for eggs of *T. trichiura*, *A. lumbricoides* and hookworm by experienced technicians [22]. A subsequent independent quality control of sample results (approximately 10%) will be conducted. All microscopically analyzed quadruplicate Kato-Katz thick smears will be destroyed within one day (after passing the quality control).

A portion of 2–3 g of stool from each specimen will be preserved in 70% ethanol and shipped to the Swiss TPH, Basel, Switzerland for PCR analysis which will allow further classification of hookworm infection into the three species *N. americanus*, *A. duodenale* and *A. ceylanicum* [23]. A subsample of 10 participants with high intensity infections following treatment from each of the settings (30 in total) will be subjected to deep sequencing for characterization of *T. trichiura* strains and investigation of potential resistance markers [24].

Additionally, a small amount of feces (less than 1 mg) from the second stool sample of participants identified as positive for *T. trichiura* in the first sample will further be tested for fecal occult blood and fecal calprotectin (Actim Fecal Blood and Calprotectin, Oy Medix Biochemica Ab, Espoo, Finland) as proxies for gut morbidity and inflammation using a rapid diagnostic immunoassay test [25]. In Lao PDR, the remains of each stool sample (ideally 10 to 20 g) will be processed by the Baermann technique for identification of *S. stercoralis* infections and be recorded qualitatively as larva-positive or negative [26].

#### Blood samples

Enrolled participants undergoing a clinical examination at baseline, 6 months and 12 months will be asked to provide a finger-prick blood sample to evaluate Hb levels using a HemoCue analyzer (Hemocue Hb 301 system; Angelholm, Sweden). Additionally, participants in Côte d’Ivoire and Lao PDR will be asked to provide a finger-prick blood sample for an RDT (i.e. Humasis Pf/Pan and Humasis Pf/Pv) for *Plasmodium* spp. infection, while participants in Côte d’Ivoire and Pemba will be asked to provide a finger-prick blood sample for an RDT (i.e. ENCODE Filariasis IgG/IgM) detecting antibodies in the blood to identify potential co-infection with *Wuchereria bancrofti*, as patients with filariasis have shown a significantly higher frequency of adverse events after combined treatment with ivermectin and albendazole [27, 28].

In one African (i.e., Côte d’Ivoire) and the Asian setting (i.e., Lao PDR), participants will be asked to give approximately 8 ml of venous blood at baseline, 6 months and 12 months to assess potential improvement on nutritional indicators for micronutrient (i.e. (pro-) vitamins, inflammation markers, and iron/ferritin) and macronutrient (i.e. albumin) deficiencies. Blood will be collected in EDTA and Serum Vacutainers (BD, Franklin Lakes, NJ, USA). In Côte d’Ivoire and Lao PDR we will undertake analysis of biochemical and hematological parameters as a proxy for functioning of vital organs. These parameters may include urea, creatinine, bilirubin, azotemia, Alanine Amino Transferase (ALAT), Aspartate Amino Transferase (ASAT), hematocrit, erythrocytes and platelets. Serum separated from collected blood in serum blood collection tubes after centrifugation will be aliquoted at local laboratories in Côte d’Ivoire and Lao PDR and then sent on dry ice to accredited reference laboratories within Switzerland and Germany. Samples will be kept at − 20 °C in field labs and in transport and stored at − 80 °C in Switzerland and in Germany. Ferritin, soluble transferrin receptor, retinol-binding protein, α1-acid glycoprotein and C-reactive protein will be measured using Sandwich enzyme-linked immunosorbent assay (ELISA) techniques [29]. Transferrin will be measured using immunoturbidimetry; iron will be measured using spectrophotometry; hepcidin will be measured using a solid phase ELISA; and vitamin A will be measured using high performance liquid chromatography.

#### Urine samples

Female participants over the age of ten years will be asked to give a urine sample of at least 10 ml for a pregnancy RDT at baseline, 6 months and 12 months before (re-)treatment is administered to avoid accidental treatment of pregnant girls/women.

### Questionnaire

#### Household questionnaire

A household questionnaire will be administered to all participating households between screening and 3 weeks follow-up to assess socioeconomic factors (e.g. structure, condition, amenities), presence of sanitation/water facilities (e.g. shower, latrine/toilet, water sources), and hygiene attitudes/practices (e.g. defecation, hand-washing, water use). Collected information will be used to assess the relationship between reinfection rates and household characteristics/behaviors; moreover, an evaluation of the potential associations of high intensity and persistent infections with sociodemographic characteristics will be conducted.

#### Treatment satisfaction

Subjective treatment satisfaction will be assessed at 3 h, 3 weeks and 6 months after treatment to investigate relationship with treatment compliance and observed efficacy in reducing egg output and morbidity. Participants will be asked to provide short-term treatment satisfaction (e.g. convenience of treatment) at 3 h and 3 weeks post-treatment, while long-term treatment satisfaction (e.g. effectiveness in reducing symptoms) will be asked at 6 months follow-up.

#### Physical functioning and well-being

Children 6 to 16 years of age will be administered a patient-rated physical functioning and well-being questionnaire during clinical examination before treatment and at 6 months and 12 months follow-up using tools already applied and evaluated in school-aged children from rural settings in Côte d’Ivoire and pre-tested in a comparable school-aged population not otherwise involved in this trial [30].

### Adverse events

Very few adverse events (AEs) are expected after ivermectin-albendazole co-administration in STH-infected individuals. The most common AEs reported were abdominal cramps, headache, fatigue, nausea, diarrhea, fever and vertigo [31–33].

Subjects will be kept for observation for at least 3 h following treatment for any acute AEs. In addition, patients will also be interviewed 3 h, 24 h, and 3 weeks after treatment about the occurrence of AEs. If there is any abnormal finding, the local study physician will perform a full clinical, physical and biochemical examination and findings will be recorded. An emergency kit will be available on site to treat any medical conditions that warrant urgent medical intervention. Information on all AEs (onset, duration, intensity, seriousness and causality) will be immediately entered in the appropriate AE module of the CRF that serves as source document. For all AEs and serious adverse events (SAEs), sufficient information will be pursued and/or obtained so as to be graded on severity, relatedness and expectedness. These data will be recorded on the appropriate CRF sections, regardless of whether they are thought to be associated with the study or the drug under investigation. Any study-related unanticipated problem posing risk of harm to subjects or others (including all unexpected adverse drug reactions) and any type of SAE will be immediately (within a maximum of 24 h after becoming aware of the event) notified to the study sponsor-investigator and co-PIs.

### Statistical methods

The primary available-case analysis will include all participants with primary endpoint data. In addition, an intention-to-treat analysis for the primary endpoint assessed at 3 weeks will be conducted considering all participants with missing endpoint data as treatment failure or all as treatment success to ensure that the results are not sensitive to potential loss to follow-up bias. CRs will be calculated as the percentage of egg-positive participants at baseline who become egg-negative after treatment. Infection intensity expressed as the arithmetic and geometric mean EPG will be calculated for each treatment arm. EPG will be assessed by adding up the egg counts from the quadruplicate Kato-Katz thick smears and multiplying this number by a factor of six. The ERR will be calculated as:

$$ ERR=1-\frac{\frac{1}{n}\ {e}^{\sum \log \left({EPG}_{follow- up}+1\right)}-1}{\frac{1}{n}\ {e}^{\sum \log \left({EPG}_{baseline}+1\right)}-1} $$

In the primary model we estimate the difference among CRs by using unadjusted logistic regressions. In a subsequent analysis an adjusted logistic regression (adjustment for age, sex and weight) will be performed.

Geometric mean egg counts will be calculated for the different treatment arms before and after treatment to assess the corresponding ERRs. Bootstrap resampling method with 5000 replicates will be used to calculate 95% confidence intervals for ERRs and the difference between the ERRs.

Results from the stool RDT for fecal occult blood will be categorized as negative, trace and positive. For calprotectin, the semiquantitative RDT allows classifying individuals by concentration into negative (levels below 50 μg/g of feces), low (50–200 μg/g) or high (≥ 200 μg/g) intensities.

Anthropometric measurements such as height and weight of school-aged children will be translated into weight-for-age, height-for-age and weight-for-height related z-scores using readily available Stata commands calculating growth indicators for children/adolescents 5–19 years [34]. Body mass index and indicators for muscle and fat tissue, such as MUAC and skinfold thickness, will serve as additional indicators of nutritional status for adults [35].

Questionnaires on physical functioning and treatment satisfaction will be evaluated by creating summary scores by summing up and transforming the single question scores according to the following formula: [(actual raw score-lowest possible raw score)/(possible raw score range)]*100 [30].

Nutritional and morbidity indicators will be analyzed using logistic and linear regression as appropriate. To compare individual’s changes in nutrition/morbidity categories as an effect from treatment, McNemar’s test will be applied. The analysis after 6 and 12 months of follow-up will be complemented by generalized estimating equation models with independent correlation structure and empirical estimators to account for missing data.

AEs will be evaluated descriptively as the difference of proportion reporting AEs before and after treatment.

## Discussion

Soil-transmitted helminthiasis remains a public health burden in many low- and middle-income countries [4]. Since the passing of the resolution WHA 54.19 by the World Health Assembly in 2001, great strides have been taken to reduce the morbidity and mortality of STH infections [36]. With the changing epidemiological landscape of soil-transmitted helminthiasis moving from control to elimination, MDA implementation is shifting from school-based to community-based [37, 38]. This trial marks the first multi-country, longitudinal, randomized double-blind controlled trial assessing the safety and efficacy of a combination therapy at the community level.

With the scaling up of PC programs, mounting drug pressure increases the risk for drug resistance against the benzimidazoles in populations infected by STHs. Nonetheless, combination treatment of two or more drugs can provide heightened efficacy and protection against drug resistance [39]. Our data will provide robust evidence on the possible increased efficacy and extended effects of combined albendazole and ivermectin treatment when compared to albendazole alone to pave the way of the former as recommended treatment for soil-transmitted helminthiasis for use in control programs.

We will report not only on efficacy and safety outcomes of combination ivermectin and albendazole therapy, but also the effects of drug administration on morbidity in a broad age range. In the midst of controversy on the impact of PC through mass deworming campaigns, this trial provides direct evidence in determining the relationship between deworming campaigns and clinical morbidity and nutritional indicators [5, 40–44]. The role of vitamin A as a protective factor against re-infection will be assessed, as well as the long-term (6 and 12 months) effects of STH therapy on anemia. Moreover, serum hepcidin levels will be quantified to determine the dynamics between iron-deficiency anemia, anemia of chronic disease and infection status/intensity. The use of fecal rapid diagnostics tests as surrogates for gut morbidity will provide a novel proof of concept between STH infection status intensity. These nutritional and gut parameters will be measured in various settings (Asia and Africa) using standardized methods at 6 and 12 months in the hopes of providing clarity to the potential impact of mass deworming in communities.

We will also report on the differences in CRs and sensitivity between the current diagnostic method recommended by the WHO (Kato-Katz smear) and DNA-based methods (qPCR). A major drawback of the Kato-Katz method is the low sensitivity for infections of low intensity [45]. As countries shift from STH control to elimination, more sensitive methods of diagnosis are needed as infection intensities lower. PCR methods offer the advantage that only single samples need to be taken and multiple infections can be detected in one reading; however, estimates of sensitivity vary and standardization of technique is needed [46, 47]. This trial will provide an opportunity to collect samples from various settings where co-infections may vary and refine the current qPCR technique.

In conclusion, this trial will aim to generate evidence to inform future WHO guidelines of STH therapy and its impact on morbidity. Currently, the combination of ivermectin and albendazole is a safe and effective treatment that is currently given to more than 500 million individuals yearly as part of the Global Programme to Eliminate Lymphatic Filariasis; and there may be opportunities to transition the program to control and elimination of soil-transmitted helminthiasis [48, 49]. However, evidence on the efficacy of the co-administration of ivermectin and albendazole against STHs from high quality RCTs is needed to confirm results and scale up combination therapy in endemic areas.

## Additional files

Additional file 1: World Health Organization Trial Registration Data Set for the efficacy and safety of IVM-ALB co-administration trial summarizing the most important trial information. (DOCX 20 kb)

Additional file 2: SPIRIT checklist for the efficacy and safety of IVM-ALB co-administration trial referring to study protocol elements within manuscript sections. (DOC 122 kb)

## Abbreviations

AE: Adverse event
CR: Cure rate
CRF: Case report form
CSRS: Centre Suisse de Recherches Scientifiques
EDTA: Ethylenediaminetetraacetic acid
ELISA: Enzyme-linked immunosorbent assay
EPG: Eggs per gram
ERR: Egg reduction rate
Hb: Hemoglobin
MDA: Mass drug administration
MUAC: Mid-upper arm circumference
NIOPH: National Institute of Public Health
PC: Preventive chemotherapy
PCR: Polymerase chain reaction
PHL-IdC: Public Health Laboratory Ivo de Carneri
RDT: Rapid diagnostic test
SAE: Serious adverse event
SPIRIT: Standard protocol items: recommendations for interventional trials
STH: Soil-transmitted helminth
Swiss TPH: Swiss Tropical and Public Health Institute
WHO: World Health Organization

## References

[1] Pullan RL, Smith JL, Jasrasaria R, Brooker SJ (2014). Global numbers of infection and disease burden of soil transmitted helminth infections in 2010. Parasit Vectors.

[2] WHO (2017). Guideline: preventive chemotherapy to control soil-transmitted helminth infections in at-risk population groups.

[3] GBD 2017 DALYs and HALE Collaborators. Global, regional, and national disability-adjusted life-years (DALYs) for 359 diseases and injuries and healthy life expectancy (HALE) for 195 countries and territories, 1990–2017: a systematic analysis for the Global Burden of Disease Study 2017. Lancet. 2018;392(10159):1859–1922. 10.1016/S0140-6736(18)32335-3 PMC6252083 30415748

[4] Jourdan PM, Lamberton PHL, Fenwick A, Addiss DG. Soil-transmitted helminth infections. Lancet. 2017. 10.1016/S0140-6736(17)31930-X 28882382

[5] Schulz JD, Moser W, Hurlimann E, Keiser J (2018). Preventive chemotherapy in the fight against soil-transmitted helminthiasis: achievements and limitations. Trends Parasitol.

[6] Moser W, Schindler C, Keiser J (2017). Efficacy of recommended drugs against soil transmitted helminths: systematic review and network meta-analysis. BMJ.

[7] Lo NC, Addiss DG, Hotez PJ, King CH, Stothard JR, Evans DS, Colley DG, Lin W, Coulibaly JT, Bustinduy AL, Raso G, Bendavid E, Bogoch II, Fenwick A, Savioli L, Molyneux D, Utzinger J, Andrews JR (2017). A call to strengthen the global strategy against schistosomiasis and soil-transmitted helminthiasis: the time is now. Lancet Infect Dis.

[8] Prichard RK, Basanez MG, Boatin BA, McCarthy JS, Garcia HH, Yang GJ, Sripa B, Lustigman S (2012). A research agenda for helminth diseases of humans: intervention for control and elimination. PLoS Negl Trop Dis.

[9] WHO. The selection and use of essential medicines: report of the WHO expert committee, 2017 (including the 20th WHO model list of essential medicines and the 6th WHO model list of essential medicines for children). In: WHO tech rep Ser. Geneva: World Health Organization. p. 2017.

[10] Palmeirim MS, Hürlimann E, Knopp S, Speich B, Belizario V, Joseph SA, Vaillant M, Olliaro P, Keiser J (2018). Efficacy and safety of co-administered ivermectin plus albendazole for treating soil-transmitted helminths: a systematic review meta-analysis and individual patient data analysis. PLoS Negl Trop Dis.

[11] Belizario VY, Amarillo ME, de Leon WU, de los Reyes AE, Bugayong MG, Macatangay BJ (2003). A comparison of the efficacy of single doses of albendazole, ivermectin, and diethylcarbamazine alone or in combinations against *Ascaris* and *Trichuris* spp. Bull World Health Organ.

[12] Speich B, Moser W, Ali SM, Ame SM, Albonico M, Hattendorf J, Keiser J (2016). Efficacy and reinfection with soil-transmitted helminths 18-weeks post-treatment with albendazole-ivermectin, albendazole-mebendazole, albendazole-oxantel pamoate and mebendazole. Parasit Vectors.

[13] Chan AW, Tetzlaff JM, Altman DG, Laupacis A, Gotzsche PC, Krleza-Jeric K, Hrobjartsson A, Mann H, Dickersin K, Berlin JA, Dore CJ, Parulekar WR, Summerskill WS, Groves T, Schulz KF, Sox HC, Rockhold FW, Rennie D, Moher D (2013). SPIRIT 2013 statement: defining standard protocol items for clinical trials. Ann Intern Med.

[14] Coulibaly JT, Fürst T, Silué KD, Knopp S, Hauri D, Ouattara M, Utzinger J, N'Goran EK (2012). Intestinal parasitic infections in schoolchildren in different settings of Côte d'Ivoire: effect of diagnostic approach and implications for control. Parasit Vectors.

[15] Wimmersberger D, Coulibaly JT, Schulz J, Puchkow M, Huwyler J, N'Gbesso Y, Hattendorf J, Keiser J (2018). Efficacy and safety of ivermectin against *Trichuris trichiura* in preschool- and school-aged children: a randomized controlled dose-finding trial. Clin Infect Dis.

[16] Palmeirim MS, Ame SM, Ali SM, Hattendorf J, Keiser J (2018). Efficacy and safety of a single dose versus a multiple dose regimen of mebendazole against hookworm infections in children: a randomised, double-blind trial. EClinicalMedicine.

[17] Laymanivong S, Hangvanthong B, Keokhamphavanh B, Phommasansak M, Phinmaland B, Sanpool O, Maleewong W, Intapan PM (2014). Current status of human hookworm infections, ascariasis, trichuriasis, schistosomiasis mekongi and other trematodiases in Lao People's Democratic Republic. Am J Trop Med Hyg.

[18] WHO (2011). Haemoglobin concentrations for the diagnosis of anaemia and assessment of severity. Vitamin and Mineral Nutrition Information System.

[19] WHO (2006). Preventive chemotherapy in human helminthiasis: coordinated use of anthelminthic drugs in control interventions: a manual for health professionals and programme managers.

[20] Guzzo CA, Furtek CI, Porras AG, Chen C, Tipping R, Clineschmidt CM, Sciberras DG, Hsieh JY, Lasseter KC (2002). Safety, tolerability, and pharmacokinetics of escalating high doses of ivermectin in healthy adult subjects. J Clin Pharmacol.

[21] Pawluk SA, Roels CA, Wilby KJ, Ensom MH (2015). A review of pharmacokinetic drug-drug interactions with the anthelmintic medications albendazole and mebendazole. Clin Pharmacokinet.

[22] Katz N, Chaves A, Pellegrino J (1972). A simple device for quantitative stool thick-smear technique in schistosomiasis mansoni. Rev Inst Med Trop São Paulo.

[23] Kaisar MMM, Brienen EAT, Djuardi Y, Sartono E, Yazdanbakhsh M, Verweij JJ, Supali T, VAN Lieshout L. Improved diagnosis of *Trichuris trichiura* by using a bead-beating procedure on ethanol preserved stool samples prior to DNA isolation and the performance of multiplex real-time PCR for intestinal parasites. Parasitology. 2017;144(7):965–74. 10.1017/S0031182017000129 PMC5471844 28290266

[24] Avramenko RW, Redman EM, Lewis R, Bichuette MA, Palmeira BM, Yazwinski TA, Gilleard JS (2017). The use of nemabiome metabarcoding to explore gastro-intestinal nematode species diversity and anthelmintic treatment effectiveness in beef calves. Int J Parasitol.

[25] Bustinduy AL, Sousa-Figueiredo JC, Adriko M, Betson M, Fenwick A, Kabatereine N, Stothard JR (2013). Fecal occult blood and fecal calprotectin as point-of-care markers of intestinal morbidity in Ugandan children with *Schistosoma mansoni* infection. PLoS Negl Trop Dis.

[26] Garcia LS, Bruckner DA (1997). Diagnostic medical parasitology.

[27] Dunyo SK, Nkrumah FK, Simonsen PE (2000). A randomized double-blind placebo-controlled field trial of ivermectin and albendazole alone and in combination for the treatment of lymphatic filariasis in Ghana. Trans R Soc Trop Med Hyg.

[28] Simonsen PE, Magesa SM, Dunyo SK, Malecela-Lazaro MN, Michael E (2004). The effect of single dose ivermectin alone or in combination with albendazole on *Wuchereria bancrofti* infection in primary school children in Tanzania. Trans R Soc Trop Med Hyg.

[29] Erhardt JG, Estes JE, Pfeiffer CM, Biesalski HK, Craft NE (2004). Combined measurement of ferritin, soluble transferrin receptor, retinol binding protein, and C-reactive protein by an inexpensive, sensitive, and simple sandwich enzyme-linked immunosorbent assay technique. J Nutr.

[30] Fürst T, Müller I, Coulibaly JT, Yao AK, Utzinger J, N'Goran EK (2011). Questionnaire-based approach to assess schoolchildren's physical fitness and its potential role in exploring the putative impact of helminth and *Plasmodium* spp. infections in Côte d'Ivoire. Parasit Vectors.

[31] Knopp S, Mohammed KA, Speich B, Hattendorf J, Khamis IS, Khamis AN, Stothard JR, Rollinson D, Marti H, Utzinger J (2010). Albendazole and mebendazole administered alone or in combination with ivermectin against *Trichuris trichiura*: a randomized controlled trial. Clin Infect Dis.

[32] Ndyomugyenyi R, Kabatereine N, Olsen A, Magnussen P (2008). Efficacy of ivermectin and albendazole alone and in combination for treatment of soil-transmitted helminths in pregnancy and adverse events: a randomized open label controlled intervention trial in Masindi district, western Uganda. Am J Trop Med Hyg.

[33] Speich B, Ali SM, Ame SM, Bogoch II, Alles R, Huwyler J, Albonico M, Hattendorf J, Utzinger J, Keiser J (2015). Efficacy and safety of albendazole plus ivermectin, albendazole plus mebendazole, albendazole plus oxantel pamoate, and mebendazole alone against *Trichuris trichiura* and concomitant soil-transmitted helminth infections: a four-arm, randomised controlled trial. Lancet Infect Dis.

[34] Vidmar SI, Cole TJ, Pan HQ (2013). Standardizing anthropometric measures in children and adolescents with functions for egen: update. Stata J.

[35] Wang Y, Chen H, Preedy V (2012). Use of percentiles and Z-scores in anthropometry. Handbook of Anthropometry. Edn.

[36] World Health Assembly: WHA54.19 Schistosomiasis and soil-transmitted helminth infections. In*.* https://www.who.int/neglected_diseases/mediacentre/WHA_54.19_Eng.pdf; 2001.

[37] Brooker SJ, Mwandawiro CS, Halliday KE, Njenga SM, McHaro C, Gichuki PM, Wasunna B, Kihara JH, Njomo D, Alusala D, Chiguzo A, Turner HC, Teti C, Gwayi-Chore C, Nikolay B, Truscott JE, Hollingsworth TD, Balabanova D, Griffiths UK, Freeman MC, Allen E, Pullan RL, Anderson RM (2015). Interrupting transmission of soil-transmitted helminths: a study protocol for cluster randomised trials evaluating alternative treatment strategies and delivery systems in Kenya. BMJ Open.

[38] Pullan RL, Halliday KE, Oswald WE, McHaro C, Beaumont E, Kepha S, Witek-McManus S, Gichuki PM, Allen E, Drake T, Pitt C, Matendechero SH, Gwayi-Chore MC, Anderson RM, Njenga SM, Brooker SJ, Mwandawiro CS. Impact, equity and cost of school-based and community-wide treatment strategies for soil-transmitted helminths in Kenya: a cluster-randomised controlled trial. Lancet Infect Dis. 2018; in press. 10.1016/S0140-6736(18)32591-1 PMC6525786 31006575

[39] Moser W, Schindler C, Keiser J. Drug combinations against soil-transmitted helminth infections. Adv. Parasitol. 2019;103:91–116. 10.1016/bs.apar.2018.08.002 30878060

[40] Andrews JR, Bogoch II, Utzinger J (2017). The benefits of mass deworming on health outcomes: new evidence synthesis, the debate persists. Lancet Glob Health.

[41] Hicks JH, Kremer M, Miguel E (2015). The case for mass treatment of intestinal helminths in endemic areas. PLoS Negl Trop Dis.

[42] Montresor A, Addiss D, Albonico M, Ali SM, Ault SK, Gabrielli AF, Garba A, Gasimov E, Gyorkos T, Jamsheed MA, Levecke B, Mbabazi P, Mupfasoni D, Savioli L, Vercruysse J, Yajima A (2015). Methodological bias can lead the Cochrane collaboration to irrelevance in public health decision-making. PLoS Negl Trop Dis.

[43] Taylor-Robinson DC, Maayan N, Soares-Weiser K, Donegan S, Garner P. Deworming drugs for soil-transmitted intestinal worms in children: effects on nutritional indicators, haemoglobin, and school performance. Cochrane Database Syst Rev. 2015;(7):Cd000371. 10.1002/14651858.CD000371.pub6 PMC4523932 26202783

[44] Welch VA, Ghogomu E, Hossain A, Awasthi S, Bhutta ZA, Cumberbatch C, Fletcher R, McGowan J, Krishnaratne S, Kristjansson E, Sohani S, Suresh S, Tugwell P, White H, Wells GA (2017). Mass deworming to improve developmental health and wellbeing of children in low-income and middle-income countries: a systematic review and network meta-analysis. Lancet Glob Health.

[45] Nikolay B, Brooker SJ, Pullan RL (2014). Sensitivity of diagnostic tests for human soil-transmitted helminth infections: a meta-analysis in the absence of a true gold standard. Int J Parasitol.

[46] Khurana S, Sethi S (2017). Laboratory diagnosis of soil transmitted helminthiasis. Trop Parasitol.

[47] O'Connell EM, Nutman TB (2016). Molecular diagnostics for soil-transmitted helminths. Am J Trop Med Hyg.

[48] Becker SL, Liwanag HJ, Snyder JS, Akogun O, Belizario V, Freeman MC, Gyorkos TW, Imtiaz R, Keiser J, Krolewiecki A, Levecke B, Mwandawiro C, Pullan RL, Addiss DG, Utzinger J (2018). Toward the 2020 goal of soil-transmitted helminthiasis control and elimination. PLoS Negl Trop Dis.

[49] Ichimori K, King JD, Engels D, Yajima A, Mikhailov A, Lammie P, Ottesen EA (2014). Global programme to eliminate lymphatic filariasis: the processes underlying programme success. PLoS Negl Trop Dis.

