# Supplementary material for: Efficacy and safety of ivermectin and albendazole co-administration in school-aged children and adults infected with Trichuris trichiura: study protocol for a multi-country randomized controlled double-blind trial
Source: BMC Infect Dis. 2019 Mar 18;19:262. doi: 10.1186/s12879-019-3882-x (PMC6421712; doi:10.1186/s12879-019-3882-x)
Supplement: Supplementary file 1 — World Health Organization Trial Registration Data Set for the efficacy and safety of IVM-ALB co-administration trial summarizing the most important trial information. (DOCX 20 kb) [file 12879_2019_3882_MOESM1_ESM.docx]

**Additional file 1. World Health Organization Trial Registration Data Set for the IVM-ALB co-administration multi-country trial.**

| **Data category** | **Information** |
| --- | --- |
| Primary registry and trial identifying number | ClinicalTrials.gov NCT03527732 |
| Date of registration in primary registry | 17 May 2018 |
| Secondary identifying numbers | Ethikkommission Nordwest- und Zentralschweiz: BASEC Nr Req-2018-00494;  Ministère de la santé et de l’hygiène publique, comité national d’éthique des sciences de la vie et de la santé: 088-18/MSHP/CNESVS-km;  Ministry of Health, National Ethics Committee for Health Research, Lao PDR: 093/NECHR;  Zanzibar Medical Research and Ethics committee: ZAMREC/0003/Feb/2018 |
| Source(s) of monetary or material support | Bill and Melinda Gates Foundation, the United States of America |
| Primary sponsor | Swiss Tropical and Public Health Institute |
| Secondary sponsor(s) | None |
| Contact for public queries | Jennifer Keiser, jennifer.keiser@swisstph.ch, +41612848218 Swiss Tropical and Public Health Institute, Basel, Switzerland |
| Contact for scientific queries | Jennifer Keiser, jennifer.keiser@swisstph.ch, +41612848218 Swiss Tropical and Public Health Institute, Basel, Switzerland |
| Public title | Efficacy and safety of IVM/ALB co-administration |
| Scientific title | Efficacy and safety of ivermectin and albendazole co-administration in school-aged children and adults infected with *Trichuris trichiura*: a multi-country randomized controlled trial |
| Countries of recruitment | Côte d’Ivoire, Lao PDR, Pemba Island (Tanzania) |
| Health condition(s) or problem(s) studied | Trichuriasis |
| Intervention(s) | - Comparator drug: 400 mg albendazole single tablet (Zentel®) and tablets of placebo at day 0 administered orally - Experimental drug: 400 mg albendazole single tablet (Zentel®) and 200µg/kg using 3mg tablets of ivermectin (Stromectol®) at day 0 administered orally |
| Key inclusion and exclusion criteria | Inclusion criteria: community members (6-60 years, minimum weight: 15 kg) infected with *T. trichiura* (at least 2/4 Kato-Katz thick smears positive and infection intensities of at least 100 eggs per gram of stool) providing written informed consent signed by the adult participant or caregiver and assent by child/adolescent.  Exclusion criteria: presence of major systemic illnesses (*e.g.* severe anaemia, clinical malaria), history of acute or severe chronic disease (*e.g.* cancer, diabetes, chronic heart, liver or renal disease), recent use of anthelmintic drug (within past 4 weeks), attending other clinical trials, known allergy to study medications (*i.e.* ivermectin and albendazole), pregnancy or lactating in the 1st week after birth, currently taking medication with known interaction (*e.g.* for ivermectin: warfarin; for albendazole: cimetidine, praziquantel and dexamethasone) |
| Study type | Multi-country, parallel group, double-blind, placebo-controlled, randomized controlled trial. Phase III |
| Date of first enrolment | 24 September 2018 |
| Target sample size | 1800 |
| Recruitment status | Recruiting: participants are currently being recruited and enrolled |
| Primary outcome(s) | Cure rate (CR) against *Trichuris trichiura* 14-21 days post-treatment |
| Key secondary outcomes | - Egg reduction rate (ERR) against *T. trichiura* - CRs and ERRs against other concomitant soil-transmitted helminth infections (i.e., *Ascaris lumbricoides*, hookworm and *Strongyloides stercoralis*) - Reinfection rates - Tolerability of treatment - Infection status assessed by polymerase chain reaction |
